# Impact of morning and rotational duties on physical health of nurses working in tertiary care hospitals of Karachi

**DOI:** 10.12669/pjms.346.16187

**Published:** 2018

**Authors:** Amjad Ali, Abdur Rasheed, Subia Naz

**Affiliations:** 1 *Mr. Amjad Ali, Institute of Nursing MS-Nursing, Lecturer, Dow University of Health Sciences Karachi, Pakistan*; 2 *Dr. Abdur Rasheed, Research Department, PhD Statistic Senior Lecturer, Dow University of Health Science, Karachi, Pakistan*; 3 *Ms. Subia Naz, Institute of Nursing MS-Nursing, Lecturer, Dow University of Health Sciences, Karachi, Pakistan*

**Keywords:** Nurse, Physical health, Shift duty, Tertiary care hospital

## Abstract

**Background & Objectives:**

In the system related to health care, shifting duties are considered essential and obligatory to make sure the stability of care in hospitals. Scheduling and shifting are the key uniqueness of shift work and nurses are mostly bounded into different schedules that facilitate 24-hour care. Our objective was to identify the impact of morning and rotational duties on physical health of nurses working in tertiary care hospitals.

**Methods:**

A total of 154 nurses from two tertiary care hospitals in Karachi were included in this study. Data were collected through the Short Form Health Servay-26 between May to June 2017. Questionnaire form consisted of five domains including Physical functioning, Role limitations due to physical health, Energy/Fatigue, Pain and General health.

**Results:**

Most of the study participants were staff nurses (66.9%) and few were head nurse and assistant head nurses 13.6% and 8.4% respectively. Nurses’ characteristics such as gender, age, educational level, designation and monthly income were found significant with duty shift with p-values 0.049, 0.007, <0.001 and 0.017 respectively. Energy/Fatigue was only domain of SF-26 which showed significant mean difference (p-value <0.001) between morning and rotational duties.

**Conclusion:**

This research concludes that nurses working in rotational duties were more prone to develop physical problem as compared to morning duties. Energy/Fatigue showed significant mean difference.

## INTRODUCTION

Currently, approximately every fifth of the global workforce is occupied in shift work, 20% of European and American workers are busy at night shifts.^1^ In modern societies, night work has been documented as mainly widespread occupational factors, which affects about 15 to 20% of the working population in North America and Europe.^2^ In the system related to health care, shifting duties are considered essential and obligatory to make sure the stability of care in hospitals. Scheduling and shifting are the key uniqueness of shift work and nurses are mostly bounded into different schedules that facilitate 24-hour care.^3^

During Shift duties and night shift duties, in particular are the most common reasons for the disturbance of circadian rhythms, causing significant alteration in biological functions and sleep pattern, which can cause a disturbance in physical and psychological functions and also affect negatively on work performance.^4^ Numerous researchers have identified the association between shift work and its impact on the physical health of workers. Researchers have identified that shift duties cause maladaptation syndrome, characterized by disturbed gastrointestinal disorders, risk of cardiovascular diseases and sleeping/waking problems.^5^ In recent times, a syndrome called “shift work disorder” has been recognized by symptoms which include disturbance of circadian rhythm, excessive day sleepiness, insomnia and fatigue.^5^,^6^

Many studies have showed that sleep pattern among nurses of night shift were considerably of poorer quality than that of fixed/morning duties and morning and evening-rotating workers.^7^ Night shift duties induce sleep deprivation which, in turn, change job performance, the daily levels of alertness and favoring fatigue.^1^,^8^ The symptoms of fatigue, including lack of energy and sleepiness, impaired concentration and feelings of discomfort were found less severe among nurses who worked during the day than others who worked night shifts.^9^

Many researches have provided evidence that fatigue is associated with night shifts duties, can add the risk of human errors, injuries and can affect negatively on patient care.^10^ Furthermore, fatigue can reduce performance level and job satisfaction, absenteeism, favoring absence due to sickness, job attrition, turn over and frequently induces to use of psychotropic drugs.^11^ Fatigue is one of the main sources of nurses’ errors in administering medication.^12^ Increase in weight was also found among nurses doing night shift.^13^ Appetite disturbance, varicose veins, altered well-being, sleep disorder, less time and less social life support were found in higher incidence among night shift duties nurses as compare to day shift duties.^14^

The results of several studies have revealed that shift workers, compared with fixed day workers with a regular routine are more prone to experience from poor sleep.^15^,^16^ Shift workers are prone to making mistakes, especially in the early hours of morning duties. However, these can affect the provision of quality of care, patient satisfaction; productivity & work. Nurses who are working in night shift duties experience physical difficulties and sleep-related problem.^17^

Nurses have a very important part in provision of quality care to patients. There are limited studies which have explored the importance of nurses’ concerns in Pakistan. The primary focus of this study was to find out the physical problems faced by nurses due to shift duties and its associated factors. More importantly, this is perhaps the first-ever study in Pakistani context on the nurses’ physical health and duty shift. The aim of this study was to identify the impact of morning and rotational duties on physical health of nurses working in tertiary care hospital.

## METHODS

A total of 154 nurses from Civil and Dow University Hospitals, Karachi participated in this study. Study participants were included after taking written informed consent and approval was taken from the Institutional Review Board (IRB) - DUHS to conduct this study. Data were collected between May to June 2017 through the Short Form Health Survey-26, which is brief 26-item questionnaire designed to detect the physical health of nurses. The questionnaire form consists of five domains including Physical functioning, Role limitations due to physical health, Energy/fatigue, Pain and General health with 10, 04, 04, 02 & 05 numbers of questions respectively. Each domain of SF-26 consists of different response categories and value. Physical functioning includes values 0, 50 &100 (1, 2, 3), Role limitation due to physical health 0 &100 (1, 2), Energy/Fatigue 100, 80, 60, 40, 20&0, and 0, 20, 40, 60, 80&100 (1, 2, 3, 4, 5, 6). Whereas, Pain includes value 100, 80, 60, 40, 20 &0 and 100, 75, 50, 25 & 0 (1, 2, 3, 4, 5, 6 and 1, 2, 3, 4, 5) and General health 100, 75, 50, 25 & 0 and 0, 25, 50, 75, 100 (1, 2, 3, 4, 5). All data were analyzed through SPSS version 21.0. Categorical variables were reported through frequencies and percentages whereas means and standard deviations were calculated for quantitative variables. Two sample independent t-tests were performed for identifying significant mean difference for each domain of SF-26 between morning and rotational duties nurses. The chi-square test of independence was also used to explore the association of nurses’ characteristics with morning and rotational duties.

## RESULTS

The distribution of nurses’ characteristics with respect to duty shift are shown in Table-I. Two third of the participants having age in between 15-25 years were performing rotation duties whereas, the same number of nurses belonged to 26 to 35 years of age were working in morning and rotation duties. This study exhibits that marital status did not influence involvement in morning or rotation duties. Graduate and postgraduate nurses more involved in morning duties as compared to an intermediate level nurse. Furthermore, experienced nurses like head and the assistant nurse were not or less involved in rotational duties. From this study, it was found that age, education level, designation and monthly income were significantly associated with duty shift pvalues ≤ 0.05.

**Table-I:** Nurses’ characteristics and duty shift.

Nurses’ characteristics		Duty Shift		Chi-square
		Morning n (%)	Rotation n (%)
Age (Years)	15-25	9 (34.6)	17 (65.4)	0.049*
	26-35	55 (50)	55 (50)	
	> 35	13 (72.2)	5 (27.8)	
Gender	Male	48 (55.8)	38 (44.2)	0.105
	Female	29 (42.6)	39 (57.4)	
Marital Status	Single	28 (41.8)	39 (58.2)	0.074
	Married	49 (56.3)	38 (48.7)	
Education level	Matriculation	10 (55.6)	8 (44.4)	0.007*
	Intermediate	12 (27.9)	31 (72.1)	
	Graduate	37 (56.9)	28 (43.1)	
	Post-graduate	18 (64.3)	10 (35.7)	
Job nature	Permanent	33 (45.2)	40 (54.8)	0.158
	Contract	35 (50.7)	34 (49.3)	
	Others	9 (75)	3 (25)	
Designation	Head nurse	21 (100)	0 (0)	<0.001*
	Assistant H/N	10 (76.9)	3 (23.1)	
	Staff nurse	38 (36.9)	65 (63.1)	
	Others (supervisor/manager)	8 (47.1)	9 (52.9)	
Monthly Income (PKR)	< 20000	7 (70)	3 (30)	0.017*
	21000-40000	47 (42.7)	63 (57.3)	
	>41000	23 (67.6)	11 (32.4)	

* p-value < 0.05 considered as significant (chi-square test)

For each domain physical functioning, role limitation due to physical health, energy/fatigue, pain, and general health there were 10, 4, 4, 2, 4 items respectively. Table-II shows a high level of internal consistency among internal items of each domain. Values of Cronbach’s reliabilities for every domain were greater than 0.7.

**Table-II:** Reliabilities analysis of SF-26 domains.

SF-26 scale	Cronbach’s α
Physical Functioning	0.909
Role Limitation due to Physical Health	0.985
Energy/Fatigue	0.708
Pain	0.846
General Health	0.713

The mean (SD) of each domain of SF-26 scale with 95% CI is described in Table-III. The highest mean score was obtained for pain 71.70 with SD 21.39, whereas lowest recorded for role limitation due to physical health 28.57 with SD 44.32.

**Table-III:** Mean and SD of five domains of SF-26 scale.

SF-26 scale	Mean	SD	95% CI
Physical Functioning	51.13	27.63	46.73--55.53
Role Limitation due to Physical Health	28.57	44.32	21.51--35.62
Energy/Fatigue	53.57	09.23	52.10--55.04
Pain	71.70	21.39	68.38--75.19
General Health	66.34	09.52	64.82--67.85

The difference of mean scores between morning and rotation duties for each domain of SF-26 scale is shown in Table-IV. P-values were obtained through a two-sample independent t-test. It is cleared from above Table-IV that smallest difference was observed for general health domain whereas largest recorded for pain, however, these differences were not statistically significant. The only domain that shows a significant difference between the mean scores of morning and rotation duties is energy/fatigue p-value < 0.001.

**Table-IV:** Mean difference of five domains of SF-26 between morning and rotation duties.

SF-26 scale	Duty shift	Mean	SD	Mean difference	95%CI	P-value
Physical Functioning	Morning	52.53	29.33	2.79	-6.02 —11.61	0.532
	Rotation	49.74	25.94			
Role Limitation due to Physical Health	Morning	31.17	45.55	5.19	-8.94 —19.33	0.469
	Rotation	25.97	43.20			
Energy/Fatigue	Morning	51.23	20.44	-4.68	-7.53— -1.82	0.001*
	Rotation	55.91	21.95			
Pain	Morning	75.06	7.53	6.56	-0.19— 13.31	0.057
	Rotation	68.51	10.19			
General Health	Morning	66.02	10.58	-0.65	-3.69— 2.39	0.674
	Rotation	66.67	8.39			

* p-value < 0.05 considered as significant (t-test).

## DISCUSSION

This research confirms the impact of rational duties on physical health. All five domains of SF-26 were reliable internally. Domains’ mean scores were compared with morning and rotation duties. Mean scores of general health for morning and rotational duties were found to be closest among all other domains whereas, pain score showed the largest mean difference. In this study fatigue/energy was significantly associated (p-value <0.001) with duty shift which was supported by the studies conducted in Italy and Brazil in 2016.^18^,^19^ Another study conducted among Norwegian nurses supported our finding that shifting duties have impact on nurses’ fatigue/Energy level.^20^

Present study has also highlighted that other domains, including physical functioning, role limitation due to physical health, pain and general health did not show significant association with morning and rotational duties. These findings were not supported by the study conducted in Egypt,^21^ which showed that night shift duty affected on pain level. Furthermore, another study conducted in Turkey found physical functioning, pain, physical health and general health as significant factors.^22^ Which is also not supported by our study.

In this study majority of the participants were younger than 35 years and study participants’ age found to significantly associated with duty shift variable (p-value = 0.049). These findings were supported by the studies conducted in Italy^18^ and Egypt^21^ in 2016. Another study accomplished among Korea health panel in 2016, found age as a significant factor between shifting and rotational duties.^23^ However, a study conducted among Tunisian nurses in 2017 did not support this finding.^24^ It was found that most of the nurses with higher education e.g., graduation/post-graduation more involved in fixed/morning duty, while nurses with relatively less education were more prevalent in rotational duties. Similar to the studies conducted in Italy, Egypt, and Korea in 2016.^21^-^23^ Our study also showed a significant association between education level and duty shift (p-value 0.007). It was observed that nurses with superior designations (supervisors/managers) prevalent in fixed/morning duty, whereas, majority of staff nurses were involved in shifting duties. Significant association of designation level (p-value <0.001) in this study is also in line with the Italian study.^18^

### Limitations of the study

As this is a cross-sectional study, it is hard to establish causality between morning and rotational duties with the physical health of nurses. Further longitudinal studies would be conducted to confirm the present conclusions. Furthermore, the sample was taken from nurses working in two public hospitals in Karachi, Pakistan; Findings can be tested in private setup, to find out the comparison in public and private setup.

## CONCLUSION

Nurses working in rotational duties were more prone to develop physical problem as compared to morning duties. Energy/Fatigue showed significant mean difference. Moreover, nurses’ characteristics such as age, education level, designation and monthly income showed significant association with duty shift.

### Authors Contribution

**AA:** Conceived Idea, Designed Research Methodology, Manuscript Writing and approval of final version of manuscript.

**AR:** Statistical Analysis, interpreted data.

**SN:** Data Collection and compilation, Literature Review, Manuscript drafting.
